# Parental Mental Health, Fathers’ Involvement and Bedtime Resistance in Infants

**DOI:** 10.1186/s13052-019-0731-x

**Published:** 2019-11-01

**Authors:** Benedetta Ragni, Simona De Stasio, Daniela Barni, Simonetta Gentile, Rosaria Giampaolo

**Affiliations:** 1 LUMSA University, P.zza delle Vaschette, 101 00193 Rome, Italy; 2 Unit of Clinical Psychology, Bambino Gesù Children’s Hospital, Piazza S. Onofrio 4, 00165 Rome, Italy; 3 Outpatient’s Unit, University Department of Pediatrics, Bambino Gesù Children’s Hospital, Piazza S. Onofrio 4, 00165 Rome, Italy

**Keywords:** Sleep, Bedtime routines, Paternal involvement

## Abstract

**Background:**

Around the age of 6 months, difficulties in settling to sleep and frequent night awakenings are generally occurring in 20 to 30% of infants. According to the transactional model parental factors can play a significant role in influencing infant sleep development. The purpose of the current study was to explore the combined effect of infants’ factors (temperament and sleep onset problems), and parental factors (parental mental health in terms of post-partum affective disorders, consistent bedtime routines and fathers’ involvement at bedtime), on infant bedtime difficulties (e.g. fussing, crying or protesting), including both maternal and paternal perspectives.

**Methods:**

Sixty Italian intact two-parent families of infants (34 boys and 26 girls) ageing from 8 to 12 months (M = 10.73, SD = 2.54) were enrolled in the study. The parents filled out self-report questionnaires to measure the aforementioned variables. To investigate which infant and parental factors predicted infants’ bedtime difficulties, two multiple linear regressions (MR), one for fathers and one for mothers, and relative weight analyses (RWA) were conducted.

**Results:**

With regard to infants’ bedtime difficulties reported by fathers (R^2^ = .35) they were explained by infant involvement in constant bedtime routines (β = −.35, *p* = .030) and paternal involvement at bedtime (β = −.45, *p* = .007). Instead infants’ bedtime difficulties reported by mothers (R^2^ = .32) were explained by minutes the child taken to fall asleep (β = .24, *p* = .04), infant involvement in constant bedtime routines (β = −.31, *p* = .01) and bedtime paternal involvement (β = −.27, *p* = .05).

**Conclusions:**

The main results of this study emphasized the protective role of consistent bedtime routines and bedtime paternal involvement in reducing infants’ bedtime difficulties perceived both from mothers and fathers. Future research could help to raise awareness and improve understanding of the familial influences on children’s sleep, providing recommendations for educating families, school professionals, healthcare providers, and the general public on risk and protective factors that could play a meaningful role in infants and children’s developing sleep patterns.

## Background

During the first year of life, children achieve two critical developmental milestones: regulation and consolidation of sleep-wake patterns [1]. However, around the age of 6 months, difficulties in settling to sleep and frequent night wakings are generally occurring in 20 to 30% of infants [2, 3], and are typically brought to the attention of paediatricians or other child-care professionals [4]. Many studies have confirmed the negative consequences of sleep disruption for children’s emotional, social, cognitive and behavioural development [5, 6], as well as for parents and family functioning [7]. According to the transactional model of development proposed by Sameroff [8] and adapted to sleep [1, 9], multiple complex connections exist between infant sleep, intrinsic infant factors (e.g. temperament) and proximal and distal contexts (e.g. family and community) [7]. Recent findings suggest that a particular role in children’s sleep development is played by parental factors [10] such as parental psychosocial functioning [11], parents’ sleep-related behaviours [4] and the quality of the relationship with their children at bedtime (e.g. emotional availability) [7, 12].

In light of these different levels of influence, the purpose of the current study was to contribute to the growing field of knowledge linking family factors to children’s sleep. Specifically, our main aim was to explore the combined effect of infants’ factors (temperament and sleep onset problems), and parental factors (parental mental health in terms of post-partum affective disorders, consistency of bedtime routines and fathers’ involvement at bedtime), on infants’ bedtime difficulties. We collected both paternal and maternal reports as measures of the key study variables. While numerous studies have examined sleep consolidation parameters, such as the number of nocturnal awakenings, we specifically focused on infants difficulties at bedtime (e.g. fussing, crying or protesting), on the grounds that this phase is likely to be strongly influenced by parental functioning and behaviours [7]. Infants may require additional parental support to regulate their arousal and emotions at bedtime and – in addition - falling asleep requires a sleep environment free of threat, where children could feel safe [13]. According to the emotional security model [14], feelings of safety and emotional security stem from stable and predictable family relationships. Children can react with emotional distress to familiar disorders and parental personal difficulties (e.g. negative affectivity disorder), suggesting parents can potentially induce feelings of stress and anxiety, which are unfavourable to sleep onset [13, 15].

In the literature, the operationalisation of sleep problems generally includes parameters related to the frequency (e.g. the number of night wakings, nights per week with bed resistance), severity and chronicity (e.g. weeks versus months) of problematic sleep behaviours [4]. Given our own focus on difficulties at bedtime, we included sleep onset issues among the potential predictor variables in our research design and, following the Diagnostic Classification of Mental Health and Development Disorders of Infancy and Early Childhood [16], operationalised these problems via sleep onset latency (the number of minutes it takes the child to fall asleep).

### Infant temperament and sleep onset difficulties

Infant sleep development is thought to be influenced by intrinsic infant characteristics such as temperament, maturation, or medical conditions [17], in addition to parental factors. Previous studies exploring temperament and sleep outcomes during the first year of life found that temperament influences total sleep duration [17–19], night wakings [27], and sleep problems [21]. Specifically, negative or difficult infant temperament, including a tendency to experience and express negative emotionality with greater intensity and frequency than other infants [22], is associated with shorter sleep duration [20, 31] and increased night wakings [27]. Children with greater negative emotionality can find it difficult to self-soothe and may rely more strongly on their caregivers for comfort and support [22]. This is are consistent with Belsky and colleagues’ differential susceptibility hypothesis [23], which posits that temperament, including negative emotionality, may render the child more susceptible to both positive or negative family environments. Temperament may also act as a moderator between parental characteristics and child sleep. For example, Netsi and colleagues [24] assessed infant reactivity as a moderator of the relationship between maternal symptoms of prenatal depression and infant sleep, finding that reactive infants had a higher number of awakenings and shorter sleep duration when exposed to maternal depression than did infants with lower reactivity. In other words, children’s internal characteristics, such as temperament, appear to be implicated in the mechanisms underlying the relationship between parental factors, child factors, and sleep quality [10].

Amongst infants’ characteristics, also sleep onset difficulties (prolonged sleep onset latency) can also influence children’s self-regulation and elicit more negative parenting at bedtime [25]: infants with sleep onset issues may require more prolonged parental presence at bedtime (which is thought to compromise infants’ ability to self-regulated their sleep), influencing parental perceptions of bedtime difficulties, such as crying or protesting.

### Parental mental health and infant sleep

Sleep onset and maintenance require a threat-free environment, feelings of safety and emotional security, all based on stable family functioning [13, 15]. According to the transactional model, parental characteristics such as personality and mental health can influence infant sleep quality, also impacting on parental bedtime and night-time practices [9]. Previous studies that took parental mental health into account have mainly focused on maternal depression, stress and anxiety as predictors of infant sleep problems [10]. Higher levels of stress and depression are negatively associated with parental warmness and reciprocity [26], which are both key contributing factors to good quality sleep in children. For example, Hughes and colleagues [27], found that mothers of 9-month old infants with shorter sleep duration and greater sleep problems displayed higher levels of parental stress and depressive symptoms. Only a few studies have also included fathers to explore the relationship between paternal mental health, children’s sleep problems and parent-child sleep interactions at bedtime [11, 27–30]. Furthermore, paternal emotional life during the transition to fatherhood and after the birth of a child has been overlooked for a long time, leaving paternal affective disorders a relatively unrecognized issue [31]. Yet, the research to date suggests that fathers can experience the same perinatal affective disorders as mothers, including post-partum depression [32]. Overall, within research on the impact of the broader family context on children’s sleep, there still appears to be a lack of studies on fathers’ role in their children’s sleep development [10].

### Bedtime routines and paternal bedtime involvement

As reported by Mindell and Williamson in a recent review on bedtime routine research [4], the regulation and consolidation of sleep-wake patterns requires the establishment and maintenance of regular sleep routines, or a set of familiar activities which parents engage their children, in the same order, every night, before lights-out [4]. The most recent studies on how bedtime routines affect sleep have shown them to promote good quality infant sleep [4]. Thus, the most immediate way for parents to positively impact on their young children’s sleep may be to implement bedtime routines [38]. Indeed, parent-child interaction of itself has a regulatory effect on child sleep quality, acting as a moderator of biological rhythms and infant self-regulation [34]. The literature suggests that infant sleep quality is related to parental features such as emotional availability, warmth, and sensitivity at bedtime. Negative parent-child relationships, in contrast, can induce a status of arousal and vigilance, producing psychological discomfort and disrupting children’s sleep.

Again, studies on how parental involvement and interaction with children affect infant sleep quality have focused more frequently in mothers than on fathers. Yet, developmental outcomes throughout childhood are known to be related to paternal bedtime involvement and the father-child relationship [33]. Several authors have reported that paternal factors may influence children’s sleep quality [9, 34]. For example, Titotzky and colleagues [34] found that greater paternal involvement was associated to fewer night awakenings at one and 6 months and Bell and Belsky [35] observed a higher rate of mother-reported sleep problems in children with absent fathers. Hence, both maternal and paternal involvement appear to play a key role in infants’ sleep quality and thus need to be further investigated to better understand parents’ influence on the quality of their children’s sleep.

## The current study

The aim of this cross-sectional study was to contribute to the growing body of knowledge about the interconnections between intrinsic infant factors, parental characteristics, bedtime routines and sleep quality. Specifically, we set out to examine, within an integrative predictive model, the relative contributions of infant factors (temperament and number of minutes typically required to fall asleep), parental mental health (post-partum affective disorders), consistent bedtime routines, and fathers’ involvement at bedtime, to infants’ bedtime difficulties (e.g. fussing, crying, protesting) as perceived by both their parents.

While past studies have tended to focus on sleep consolidation parameters, such as an infant’s mean number of nocturnal awakenings, we particularly focused on infants difficulties at bedtime (e.g. fussing, crying or protesting), expecting this time of day to be highly influenced by parental functioning and behaviours [7]. Bedtime is a time when most caregivers are present and can interactively engage with their children [4]. Indeed, infants may require more parental support to regulate their arousal and emotions at bedtime, while falling asleep demands a non-threatening sleep environment where children can feel safe [13]. A number of authors have pointed to the role of daytime, as well as bedtime parenting quality and parental strategies in predicting sleep disruptions in early childhood [36, 37]. Studies with direct observations of maternal behaviours at bedtime showed that more emotionally available mothering predicted fewer mother-reported infants’ night awakenings [12]. Quality mother-infant interactions and activities are associated with reduced night-time distress and better quality of sleep in early infancy [7, 38].

To the best of our knowledge, this is the first study that has explored the relationships between infant temperament, parental post-partum affective disorders (e.g. post-partum depression), consistency of bedtime routines, and paternal involvement in bedtimes. We integrate all of these variables into a novel predictive model of infants’ bedtime difficulties, gathering the relevant data from both mothers and fathers.

Specifically, both parents were asked to provide information about their child’s temperament, sleep-related behaviors and their post-partum affective symptoms, and the father’s involvement in the infant’s bedtime care.

We hypothesized that higher levels of negative emotionality and longer sleep onset latency in the child, and post-partum affective symptoms in the parents would predict infant bedtime difficulties. On the contrary, we expected that constant bedtime routines and paternal bedtime involvement would be associated with fewer bedtime difficulties as perceived by both mothers and fathers.

## Methods

### Participants and procedure

Sixty Italian intact two-parent families of infants (34 boys and 26 girls) ageing from 8 to 12 months (M = 10.73, SD = 2.54) were enrolled in the study. Maternal mean age was 34.10 years (SD = 5.04) and paternal one was 36.25 years (SD = 5.48). Criteria for participation were full-term pregnancy, the absence of hospitalisations lasting more than a week and the absence of any physical or mental disability.

The parents, who gave written informed consent for participation in the study, filled out anonymous self-report questionnaires at home. They were invited to fill out the questionnaires independently and were each provided with a prepared envelope for return of the questionnaires. Mothers were also asked to provide information about demographic characteristics of the family and the child’s medical and developmental history. The study was approved by the Ethics Committee for Scientific Research of LUMSA University of Rome, Italy and followed the APA ethical guidelines of research.

### Measures

#### The expanded version of the brief infant sleep questionnaire (BISQ [39])

Both mothers and fathers completed the BISQ, a self-report measure including specific questions about infant nighttime sleep patterns, sleep-related behaviors, sleeping arrangements, bedtime routines, and other parental interventions. Parents were asked to describe their children’s behaviors during the last 2 weeks. As in other studies [4, 40, 41], we selected the following BISQ items as indicators of sleep difficulties: “Typically, how difficult is bedtime for your child, for example, fussing, crying, protesting?”; “How long does it typically take your child to fall asleep at night?”; “In a typical 7-day week, how often does your child have the exact same bedtime routine?”. The BISQ has been validated against actigraphy and daily-logs with significant correlations between sleep onset (*p* < .001 for both), sleep duration (*p* < .05 for both) and night wakings (*p* < .0001) [42]. Moreover, its sensitivity in documenting infant sleep and the effects of environmental factors has been well established [39].

By an ad-hoc item, paternal and maternal perceptions about father’s involvement in bedtime children’s caring was also evaluated (for fathers: “How much do you take care of child at bedtime?”; for mothers: “How much does your partner take care of child at bedtime?”). The item was rated on a 5-point Likert scale and the higher was the score, the higher was perceived fathers’ involvement at bedtime.

#### Negative emotionality from Italian temperament questionnaires (QUIT version 1–12 months [43])

Both parents filled the Negative Emotionality subscale from the Italian Temperament Questionnaire, which is composed of 8 items rated on a 6-point Likert scale (1 = almost never to 6 = almost always) and describes infant behavior in 3 different contexts: (1) child interaction with others, (2) child during play, and (3) child during an activity or a task (e.g. “when you hold your baby he/she cries”; when the mother get away the baby, he/she cries out loud”; while he/she is playing has an angry or sad expression”). The final score was averaged to create Negative Emotionality total rate for each infant. For the current study, we selected the Negative Emotionality dimension as predictors of infants’ bedtime difficulties according to findings of researchers investigating the relationship between infant temperament and sleep problem [23, 27, 30, 31]. The higher was the score, the higher was the child’s negative emotionality reported by parents. Cronbach’s alpha was.76 for fathers and .64 for mothers.

#### Perinatal assessment of paternal and maternal affectivity (PAPA and PAMA [44])

PAPA and PAMA are self-report measures of a series of psycho-social and physical dimensions related to affectivity, as experienced by a father and mother in the last 2 weeks, in the period from pregnancy to the 1st year after the birth of the infant. Both are composed of 11 items describing problems rated in terms of severity (“Not at all”, “A bit”, “Moderately and “A lot”). The first eight items deal with the following dimensions: Anxiety, Depression, Perceived Stress, Irritability/Anger (irritability, hostility, arguments with others, anger attacks), Relationship Problems (including couple, family, friends and at work), Abnormal Illness Behavior (somatization, functional medical syndromes, chronic pain syndromes, hypochondriac complaints), Physiological problems (with sleeping eating or sexual desire), Addictions (smoking, drinking alcohol, taking drugs, gambling, compulsive use of the Internet) and other Risky Behaviors (such as driving at high speed, dangerous sports or taking unnecessary risks at work). The last three items ask questions relating to fatherhood and motherhood and cultural factors. The questions are: “Do you think your answers to these questions are related to being, or becoming, a father/mother? If “YES” or “Possibly”, in what way?”; “Do you feel happy or content with being, or becoming, a father/mother?” and “Are there other questions, or words, that would be better to describe how you have been feeling over the past two weeks? If “YES”, please describe”. The purpose of this questionnaire is not to make a diagnosis of postpartum depression, but to identify fathers/mothers showing a significant risk in this regard. Cronbach’s alpha was.87 for fathers and .88 for mothers.

## Data analyses

### Preliminary analysis

Data were analysed using SPSS software version 24.0 (Armonk, NY: IBM Corp). Preliminarily, we described the study variables in terms of means, standard deviations and ranges (Tables 1 and 2.).

**Table 1:** Bivariate correlations between BISQ, QUIT, PAPA, PAMA, paternal involvement and bedtime difficulties perceived by fathers

	1	2	3	4	5	6	7
1. Infants’ Bedtime Difficulties_BISQ_FA	1						
2. Negative Emotionality_QUIT_FA	.21	1					
3. Minutes taken to fall asleep_BISQ_FA	.36**	.13	1				
4. Paternal Post-partum affective disorders PAPA_TOT	.25*	.38*	.27*	1			
5. Maternal Post-partum affective disorders PAMA_TOT	.29*	.38*	.23	.45**	1		
6. Constant Bedtime Routines_BISQ_FA	−.11	.33*	.07	.01	−.02	1	
7. Paternal Involvement_FA	−.37**	.06	−.24	.07	.04	−.15	1
Mean	2.35	2.76	2.58	6.01	6.75	4.27	3.27
SD	1.20	.73	.69	4.43	4.67	.91	.86
Range	1–5	0–4.3	1–4	0–16	0–21	1–5	2–5

* *p* < .05; ** *p* < .01

**Abbreviations:** *FA* = reported by fathers; *BISQ* = Brief Infant Sleep Questionnaire; *QUIT* = Italian Temperament Questionnaires; *PAPA_TOT* = Perinatal Assessment of Paternal Affectivity Total score; *PAMA_TOT* = Perinatal Assessment of Maternal Affectivity Total score

**Table 2:** Bivariate correlations between BISQ, QUIT, PAPA, PAMA, paternal involvement and bedtime difficulties perceived by mothers

	1	2	3	4	5	6	7
1. Infants’ Bedtime Difficulties_BISQ_MO	1						
2. Negative Emotionality_QUIT_MO	.04	1					
3. Minutes taken to fall asleep_BISQ_MO	.30*	.10	1				
4. Paternal Post-partum affective disorders PAPA_TOT	.27*	.01	.08	1			
5. Maternal Post-partum affective disorders PAMA_TOT	.32*	.14	.17	.45**	1		
6. Constant Bedtime Routines_BISQ_MO	−.37**	.02	−.15	−.12	−.13	1	
7. Paternal Involvement_MO	−.15	−.52*	.02	−.03	.06	−.12	1
Mean	2.10	2.73	2.44	6.01	6.75	4.34	3.18
SD	1.27	.70	.74	4.43	4.67	1.06	1.01
Range	1–5	0–4.5	1–4	0–16	0–21	1–5	1–5

* *p* < .05; ** *p* < .01

**Abbreviations:** *MO* = reported by mothers; *BISQ* = Brief Infant Sleep Questionnaire; *QUIT* = Italian Temperament Questionnaires; *PAPA_TOT* = Perinatal Assessment of Paternal Affectivity Total score; *PAMA_TOT* = Perinatal Assessment of Maternal Affectivity Total score

Associations between infants’ bedtime difficulties (BISQ), negative emotionality (QUIT subscale), minutes taken by the child to fall asleep (BISQ), post-partum affective disorders (PAPA and PAMA total score), infants’ involvement in constant bedtime routines (BISQ), paternal involvement at bedtime (ad-hoc question) were measured by bivariate Pearson correlations separately for fathers and mothers.

### Explaining infants’ bedtime difficulties

To investigate whether and the extent to which infants’ factors (i.e., child’s negative emotionality, minutes taken by the child to fall asleep, involvement in constant routines), parents’ post-partum affective disorders and paternal bedtime involvement predicted infants’ bedtime difficulties, we conducted two multiple linear regressions (MR), one for fathers and one for mothers, and relative weight analyses (RWA). Using MR, we estimated the overall R^2^ and determined the statistical significance of each regression coefficient. However, when predictors are correlated, such as in the case of variables refer to members of the same family, MR is not enough to adequately divide variance among the predictors because it is not able to take appropriately into account the shared contribution of correlated predictors [45, 46]. That is, the results relying solely on β inspection may be misleading. For this reason, we decided to extended MR with RWA.

RWA uses a variable transformation approach to address the issue of correlated predictors and focuses on the proportionate contribution each predictor makes to R^2^, taking into account both its unique relationship with the criterion and its relationship when combined with other predictors (relative contribution) [47]. The relative contribution can be estimated by creating a set of variables that are highly related to the original one but are uncorrelated with each other. The criterion variable can then be regressed on the new uncorrelated variables to approximate the relative weights of the original variables. The importance weights provided by the analysis can then be scaled in the metric of relative effect size by dividing the relative weights by the model R^2^ and then multiplying these values by 100. In this way, the rescaled weights are interpreted as the percentage of predicted criterion variance attributed to each predictor.

## Results

Pearson bivariate correlations between study variables are reported in Table 1 (fathers) and Table 2 (mothers). Infants’ bedtime difficulties reported by fathers were positive correlated to minutes the child taken to fall asleep (*r* = .36, *p* < .01), paternal (*r* = .25, *p* < .05) and maternal (*r* = .29, *p* < .05) post-partum affective disorders. Moreover, infants’ bedtime difficulties reported by mothers were positive correlated to minutes the child taken to fall asleep (*r* = .30, *p* < .05), paternal (*r* = .27, *p* < .05) and maternal (*r* = .32, *p* < .05) post-partum affective disorders. On the other hand, infants’ bedtime difficulties reported by fathers correlated negatively to paternal bedtime involvement (*r* = −.37, *p <* .01) and infants’ bedtime difficulties reported by mothers were negative correlated to infants’ involvement in constant bedtime routines (*r =* −.37, *p <* .01).

Moreover, significant correlations also emerged. For fathers, children’s negative emotionality was positive correlated to infants’ involvement in constant bedtime routines (*r =* .33, *p <* .05), and also to paternal (*r* = .38, *p* < .05) and maternal (*r* = .38, *p* < .05) post-partum affective disorders. Furthermore, minutes the child taken to fall asleep, reported by father, were positive correlated to paternal post-partum affective disorders (*r* = .27, *p* < .05). For mothers, children’s negative emotionality correlated negatively with paternal involvement at bedtime (*r* = −.52, *p* < .05) and finally, a positive correlation between paternal and maternal post-partum affective disorders emerged (*r* = .45, *p* < .01).

Table 3 e Table 4 report the regression and relative weight analysis results for fathers and for mothers. With regard to infants’ bedtime difficulties reported by fathers, MR predictors yielded a R^2^ = .35 and as the inspection of β coefficients suggests, infant bedtime difficulties were explained by infant involvement in constant bedtime routines (β = −.35, t(58) = − 2.228, *p* = .030) and paternal involvement (β = −.45, t(58) = − 2.975, *p* = .007).

**Table 3:** Explaining infants’ bedtime difficulties perceived by fathers

	Infants’ bedtime difficulties perceived by fathers (*R*^2^ = .35)				
Variables	MR			RWA	
	β	t	p	Raw importance	Rescaled importance
Negative Emotionality_QUIT_FA	.22	1.383	.18	.04	10.2%
Minutes took to fall asleep_BISQ_FA	.20	1.268	.21	.08	21.5%
Paternal Post-partum affective disorders PAPA_TOT	.12	.703	.49	.04	12.5%
Maternal Post-partum affective disorders PAMA_TOT	.20	1.040	.31	.03	7.9%
Constant Bedtime Routines_BISQ_FA	−.35	−2.228	.03	.03	8.9%
Paternal Involvement_FA	−.45	−2.975	.00	.14	39%
*R* ^2^				.35	100%

**Abbreviations:** *FA* = reported by fathers; *BISQ* = Brief Infant Sleep Questionnaire; *QUIT* = Italian Temperament Questionnaires; *PAPA_TOT* = Perinatal Assessment of Paternal Affectivity Total score; *PAMA_TOT* = Perinatal Assessment of Maternal Affectivity Total score

Rescaled importance was computed by dividing the relative weights by the total R2 and multiplying by 100

**Table 4:** Explaining infants’ bedtime difficulties perceived by mothers

	Infants’ bedtime difficulties perceived by mothers (*R*^2^ = .32)				
Variables	MR			RWA	
	β	t	p	Raw importance	Rescaled importance
Negative Emotionality_QUIT_MO	−.13	−.968	.34	.01	1.3%
Minutes took to fall asleep_BISQ_MO	.24	2.017	.04	.07	20.4%
Paternal Post-partum affective disorders PAPA_TOT	.18	1.351	.18	.05	17.2%
Maternal Post-partum affective disorders PAMA_TOT	.15	1.193	.24	.04	13.2%
Constant Bedtime Routines_BISQ_MO	−.31	−2.613	.01	.12	37.3%
Paternal Involvement_MO	−.27	−1.978	.05	.03	10.6%
*R* ^2^				.32	100%

**Abbreviations:** *MO* = reported by mothers; *BISQ* = Brief Infant Sleep Questionnaire; *QUIT* = Italian Temperament Questionnaires; *PAPA_TOT* = Perinatal Assessment of Paternal Affectivity Total score; *PAMA_TOT* = Perinatal Assessment of Maternal Affectivity Total score

Rescaled importance was computed by dividing the relative weights by the total R2 and multiplying by 100

The more the child and his/her father were involved in constant bedtime routines, the fewer were the child’s difficulties at bedtime (fussing, crying, protesting).

Instead for infants’ bedtime difficulties reported by mothers, MR predictors yielded a R^2^ = .32. As the inspection of β coefficients suggests, infant bedtime difficulties were associated explained by minutes the child taken to fall asleep (β = .24, t(58) = 2.017, *p* = .04), infant involvement in constant bedtime routines (β = −.31, t(58) = − 2613, *p* = .01) and paternal involvement (β = −.27, t(58) = − 1.978, *p* = .05). The more mothers involved their child in a bedtime routine every night and reported fewer minutes to put their child to sleep, the less they perceived their infants having difficulties at bedtime (fussing, crying, protesting). Moreover, the higher was the perceived paternal involvement, the lower were the child’s bedtime difficulties reported by mothers.

Regarding infants’ bedtime difficulties reported by fathers, RWA results confirmed the great importance of paternal involvement at bedtime. Paternal perceived involvement accounted for 39% of the explained variance for infant bedtime difficulties. Moreover, from this analysis it emerged more substantial contributions of minutes taken by the child to fall asleep (accounting for 21.5% of the explained variance) and of negative emotionality (accounting for 10.2%) compared to that emerged from MR.

Considering infants’ bedtime difficulties as reported by mothers, RWA results confirmed the great importance of infant involvement in constant bedtime routines (37.3%) and minutes to fall asleep, which together accounted for 57.7% of the explained variance for infant bedtime difficulties. In addition, paternal affective disorders, not significant from MR, accounted for 17.2% of the explained variance for mothers’ reports of infant bedtime difficulties, more than did maternal post-partum affective disorders (13.2%).

## Discussion

The main aim of this study was to contribute to the growing body of knowledge concerning the interrelations between intrinsic infant factors, dimensions of parenting, bedtime routines and sleep quality in the first year of life. The combined use of MR and RWA allowed us to assess the specific contribution of each variable in explaining infants bedtime difficulties (fussing, crying, protesting) perceived by both mothers and fathers, taking into account the their statistical interdependence.

In relation to our hypotheses, the results confirmed longer sleep onset latency in children as a risk factor for infant bedtime difficulties as perceived by mothers. On the contrary, consistent bedtime routines and paternal bedtime involvement were associated with fewer bedtime difficulties, as reported by both for mothers and fathers.

Hence, our first main finding was that the number of minutes it took infants to fall asleep was positively associated with bedtime difficulties as measured by maternal report. In other words, the more minutes a child required to settle down to sleep, the more the mother was likely to perceive him/her as having difficulties at bedtime. According to literature, sleep problems such as bedtime resistance may influence children’s self-regulation and specifically their capacity to regulate negative feeling in the context of parent-child interaction. This in turn may elicit more negative parenting strategies, setting off a reciprocal negative cycle [34]. More specifically, infants who are inclined to express negative emotionality at lower thresholds with greater intensity and frequency may rely more strongly on their caregivers for soothing, support, and comfort [30], influencing parental perceptions of bedtime difficulties, such as crying or protesting.

A second main finding of the current study was that consistent bedtime routines with paternal involvement protected children against the risk of bedtime difficulties as perceived by both their parents. In other words, the more parents engaged their child in a nightly bedtime routine and the more fathers were involved in their children’s bedtime care, the lesser the chance that either parents would report bedtime difficulties. This is in line with previous cross-sectional, longitudinal, and intervention studies showing that consistent implementation of a bedtime routine is associated with positive sleep outcomes including decreased sleep onset latency and enhanced caregiver-reported sleep quality [4, 48]. For example, a study of Mindell and colleagues [48] on 10,085 children ages 0 to 5 years and from 14 different countries showed that having a regular bedtime routine was associated with earlier bedtimes, shorter sleep onset latency, longer night time sleep duration, fewer night awakenings and decreased caregiver-perceived child sleep problems. In general, bedtime is a time when caregivers can engage interactively with their children and adaptive bedtime routines could generate rich opportunities for supporting positive developmental outcomes [4, 49].

With regard to the involvement of fathers, a number of studies have examined the relationship between paternal factors and children’s sleep patterns [9, 38, 50]. For example Millikovsky and colleagues [40] observed that paternal involvement can be a source of emotional and instrumental support for the mothers and is associated with moderately lower levels of maternal stress: more specifically, the fathers of children with sleep disturbances are less involved in caring for their infants, while the mothers of children with sleep disturbances reported higher levels of stress but only when paternal involvement in child caregiving was lower. Bernier [38] and Tikotzky [50] also found paternal involvement enhanced the sleep consolidation of both children and mothers. Furthermore, paternal contributions to infant care benefits mothers, by allowing them to share the effort and fatigue involved in everyday caretaking. According to Peltz [51] if each parent prevents the other from becoming overburdened and frustrated, this has a positive impact on the child’s wellbeing and facilitates the maintenance of a safe and secure environment. Couples who share infant caregiving during the day report higher levels of support and lower levels of stress; these factors can exert a calming and positive influence on the familial environment, fostering the attainment of consolidated sleep patterns [9].

From our results emerged also that paternal affective disorders, not significant from MR, accounted for 17.2% of the explained variance for mothers’ reports of infant bedtime difficulties, more than did maternal post-partum affective disorders (13.2%). Previous sleep studies that took parental mental health into account have mainly focused on maternal depression, stress and anxiety as predictors of infant sleep problems [10]. Higher levels of stress and depression are negatively associated with parental warmness and reciprocity [36], which are both crucial factors in caregiver-child interactions at bedtime and in general for good quality sleep in children. Only a few studies have also involved fathers by exploring the relationship between paternal parenting stress, children’s sleep problems and parent-child sleep interactions at bedtime [11, 29, 30]. The existing research suggests that fathers experience similar perinatal affective disorders to mothers, such as, for example, post-partum depression [31, 32]. Overall, there is still a lack of research into how fathers contribute to their children’s development of sleep patterns ad behaviours [10]. It follows that this findings should be interpreted with caution and needs to be corroborated and further explored in future studies, which should also include clinical samples, such as parents who have been diagnosed with post-partum depression.

Concerning infants’ temperament, from our results emerged that infant negative emotionality, not significant from MR, accounted for 10.2% of the explained variance for fathers’ reports of infant bedtime difficulties. Previous research [22, 23] have found negative emotionality to be related to children bedtime difficulties perceived by parents, this results, however, should be interpreted with caution and future research will need to verify and assess more broadly.

This study presents several weaknesses that call for careful interpretation of the results while suggesting new perspectives for further research. First, the sample size precluded us from conducting more sophisticated analyses such as simultaneous evaluation of all direct and interactive effects of the predictors. Future research with a sample large enough to test a causal model may bring to light a far more complex picture of the relationship between bedtime routines and parents’ psychosocial functioning. Furthermore, introducing an objective sleep measure such as actigraphy or polysomnography could provide a more reliable assessment of sleep quality, enabling more in-depth investigation of the links between parental factors and overall infant sleep development. Finally, our study was cross-sectional in nature, limiting the strength of the inferences that may be drawn from our findings.

## Conclusions

The main aim of the current study was to contribute to the growing field of knowledge linking family factors to children’s sleep, studying together infant and parental factors and their relationship with infant sleep difficulties. Specifically, the main results of this study emphasized the protective role of constant bedtime routines and paternal bedtime involvement in reducing infant bedtime difficulties perceived both from mothers and fathers. Following a bedtime routine is associated with positive sleep outcomes including decreased sleep onset latency and enhanced caregiver-reported sleep quality. Moreover, couples who share infant caregiving are characterized by higher levels of support and lower levels of stress exerting a calming and positive influence on the familial environment, promoting achievement of consolidated sleep patterns. As emerged both from the 74th Congress of the Italian Society of Pediatrics [52] and the Consensus Statement of the American Academy of Sleep Medicine [5] healthy sleep requires an adequate duration, appropriate timing, good quality and regularity. Future research could help to raise awareness and improve understanding of the familial influences on children’s sleep, providing recommendations for educating families, school professionals, healthcare providers, and the general public on risk and protective factors that could play a meaningful role in infants and children’s developing sleep patterns.

## Data Availability

Filled questionnaires are available from BR, on request.

## Abbreviations

BISQ: Brief Infant Sleep Questionnaire
FA: reported by fathers
MA: reported by mothers
MR: multiple linear regressions
PAMA_TOT: Perinatal Assessment of Maternal Affectivity Total score
PAPA_TOT: Perinatal Assessment of Paternal Affectivity Total score
QUIT: Italian Temperament Questionnaires
RWA: relative weight analyses
